# Intention to Purchase Foods Based on Insects, Arachnids, and Arthropods, Processed by 3D Printing in Panama Consumers

**DOI:** 10.1155/2024/9094666

**Published:** 2024-10-15

**Authors:** Marcos E. González-Guzmán, Shyla Del-Aguila-Arcentales, Aldo Alvarez-Risco, Mercedes Rojas-Osorio, Jaime A. Yáñez

**Affiliations:** ^1^Escuela de Posgrado, Universidad Internacional Iberoamericana, Campeche, Mexico; ^2^Facultad de Ingeniería y Tecnología, Universidad Santa María La Antigua, Ciudad de Panamá, Panama; ^3^Sustainability and Business Research Group, Escuela de Posgrado, Universidad San Ignacio de Loyola, Lima, Peru; ^4^Facultad de Administración y Negocios, Universidad Tecnológica del Perú, Lima, Peru

**Keywords:** 3D printing, entomophagy, food safety, intention to purchase, nutrition, sustainability

## Abstract

Currently, food access has worsened during the COVID-19 pandemic. For this reason, various alternatives are required to improve the population's diet. Among the many alternatives is the use of 3D printing technology to reproduce food that can reach the most vulnerable population. This remarkable study shows future generations the importance of seeking innovative food that guarantees a nutritious and accessible diet. The study focuses on the Panamanian population to determine which variables influence the decision to consume innovative foods. The innovative product to be tested is based on insects, arachnids, and arthropods, which may be difficult for the population to consume, but thanks to 3D printing technologies, it is possible to generate foods based on these raw materials that look like traditional foods. Likewise, processing these foods generates less water consumption, giving them an ecological attribute. The present study seeks to know the variables that determine the purchase intention of consumers in Panama regarding the food supply based on insects, arachnids, and arthropods that are transformed into traditional food formats using 3D printers. This information can help companies prepare food offers to consumers in Panama.

## 1. Introduction

By 2030, more than nine billion human beings have to be fed. Food can be influenced by five domains [1]: availability, socialization, literacy, marketing, and politics. It has been possible to demonstrate the negative impact of COVID-19 on food [2–4] that leads to poor nutrition and diseases due to poor nutrition. It has been possible to identify the need for social protection against hunger caused by COVID-19 [5, 6] since there are various problems with the population's access to food [1], which is a serious but hidden problem in the population [7]; for this reason, the application of technology in the automation of food production processes is required to generate alternatives for the population in these times of crisis. Thus, contributing to Sustainable Development Goals (SDGs) 1, 2, and 3, the food alternative is presented using insects, arachnids, and arthropods as raw materials [8], a viable alternative to guarantee food security for a large part of the population. The 3D printing of these foods allows them to have characteristics that make them look more appetizing, which is a key element for their market acceptance by consumers [9]. There is some research regarding the acceptance of insect, arachnid, and arthropod-based foods [10–13], but this research is the first in Panama's consumers.

3D printing and insect-based foods can address food security and SDGs. 3D printing allows personalized production of nutritious foods, reducing waste. Insects, rich in proteins, require fewer resources than traditional livestock farming, reducing environmental impact. Integrating these technologies can improve food availability, reduce the ecological footprint, and promote efficient agricultural practices, contributing to zero hunger, responsible production and consumption, and climate action SDGs. This study contributes to the SDGs as it is accompanied by the recently published Food Security and Nutritional National Plan, which sets out strategies to reduce micronutrient deficiencies and halt the increase in overweight and obesity as well as to improve agriculture's contribution to food security and reduce undernourishment.

The importance of this study is based on the need to evaluate aspects that contribute to food security in Panama since problems have been reported in the population [14, 15]. In current academic literature, there is a significant gap in the study of Panamanian consumers' attitudes towards insect-based foods processed using 3D printing. Specifically, it is necessary to know the effect of ecological concern on attitude to assess whether it is necessary to increase this concern in people through specific messages; similarly, it is relevant to know the effect of concern for safety on attitude, and it is also important to confirm whether concern for a healthy lifestyle also has an effect on attitude towards this type of food. Finally, the model will allow us to verify whether attitude has an effect on the purchase intention of consumers in Panama. It will also be helpful to confirm the mediating effect of attitude. Despite increasing global interest in sustainability and food innovations, research on the perceptions and acceptance of these foods in Panama is limited. Most studies have focused on developed markets, neglecting consumers' perspectives in emerging regions such as Latin America. This lack of data limits the understanding of potential cultural, economic, and social barriers that could influence the acceptance of these innovative products. Addressing this gap is crucial to designing effective strategies that promote food sustainability and technological innovation in the Panamanian context.

## 2. Literature Review

The problem of food, further complicated by COVID-19 [16–18], gives rise to alternatives for generating food that may be within reach of the population, be ecological, and be at an affordable price. Thus, Lozada [19] developed a bread snack incorporating *Alphitobius diaperinus* flour. Five samples of breadsticks were presented to the tasters with different percentages of *A. diaperinus* flour, from 0% to 10%, increasing the quantity by 2.5% each time. Although breadsticks with higher percentages of insect meal were used in other trials, a maximum of 10% was taken due to the strong influence of insect meal on flavor and appearance. Two sensory tests were performed: a hedonic scale preference test and a preference ranking test. It is pointed out that after having analyzed the results, the substitution of wheat flour for *A. diaperinus* flour caused a decrease in the height of the bread snacks (except in the 12.5% test) and their firmness, giving rise to a more brittle product with less volume. In addition, as the proportion of insect flour increased, the dough's color and the final product were darker, which caused the tasters to find the product less attractive when asked about its appearance. As the percentage of *A. diaperinus* flour increased, the evaluation by the judges was lower, although in no parameter was a mean lower than 3 (on a scale of 7) obtained. On the other hand, the response from potential consumers was not as negative as expected, considering that insects are not part of the traditional Western diet but are normally associated with pests and diseases. The sensory attributes most affected by incorporating the insect meal were the taste and, above all, the aftertaste or residual taste. Numerous sensory attributes significantly discriminated between insect species, and taste drivers and sensory attributes associated with food pairings were identified; in addition, males were found to associate insects with emotions such as calm and wildness, while females associated insects with joy and pleasure [20]. Most studies with whole insects and insect meal highlight that insect-based products are evaluated more negatively than control products. Although the sensory properties of insects are affected by species and processing conditions, they are generally negative in all sensory dimensions [21].

Kuff et al. [22] in Brazil assessed 194 consumers, finding that country-of-origin labeling through quality expectation (0.41) had an effect on the intention to buy insect-based foods (0.75, which was higher than the direct effect of country-of-origin labeling on the intention to buy insect-based foods (0.21)). This study assesses information and quality but does not assess consumer concerns and attitudes. Sogari et al. [23] in Italy evaluated 565 consumers and found that providing more information about the benefits of using insects in food leads to their consumption. Intention to pay for these foods is directly affected by previous experience of entomophagy. Despite this, environmental and food safety concerns remain to be assessed. Wang and Park [24] found in 219 consumers in Japan that anthropomorphism in packaging has an effect on purchase intention for insect foods. Furthermore, the effect of anthropomorphism on purchase intention was mediated by the perception of psychological closeness to insect foods. This model could be complemented by assessing environmental concern and food safety as predictors of intention to purchase insect-based foods.

Along these same lines, Zaragozano [25] shows that the intake of insects and other arthropods is increasing in countries in which this type of food was unknown or repudiated, such as in the countries of the European Union. The administration's intake of this “new food” required strict hygienic and sanitary control, from breeding the insect to reaching the consumer. Arthropods could replace traditional foods in various parts of the world in cases of occasional famine, droughts, wars, etc. Breeding insects and other arthropods in well-controlled farms guarantees a great source of high-biological value proteins and is relatively cheap for human consumption. More recently, Alvarez Miguel [26] developed and evaluated a bar with cricket flour (*Acheta domesticus*) as the primary protein source to obtain a food product with a high protein content based on this flour. During the study, three formulations of bars were carried out, substituting 10%, 15%, and 20% of the components for cricket flour. The parameters evaluated were humidity, ashes, dimensions, the weight of the piece, texture, and color in the elaborated bars. The design of three formulations of protein bars with cricket flour has been successfully developed at the laboratory level. An elaboration process was optimized using the available technology. All formulations of protein bars based on cricket flour could be designated based on regulation (EC) No. 1924/2006 as “high protein content” and also “high fiber content.” The cricket flour and the bars obtained were characterized. It can be affirmed that although the hardness of the bars has been observed when increasing the concentration of cricket flour, this trend did not show significant differences due to the great variability in pieces.

FAO [27] mentions that the habitant's growth, urbanization, and the increase of the middle class have generated more demand for food globally, especially for animal protein sources. The survival of the human species must expand through alternative sources. Eating insects complements the diet of approximately 2000 million people, and it is a habit that has always been present in eating behavior. Entomophagy is practiced in many countries worldwide, but mainly in regions of Asia, Africa, and Latin America. Several studies have shown that confidence [28] and the risk–benefit balance [29], images on packaging [30], and even marketing [31] have an effect on purchase intention, so understanding could be completed by assessing environmental concern and concern for healthy eating. Despite the previous data, Mulazzani et al. [32] found that environmental awareness had no effect on the intention to purchase food-based foods.

## 3. Theoretical Framework and Hypotheses

### 3.1. Theory of Planned Behavior (TPB)

The first publication of Ajzen's TPB was in 1985 [33], becoming a seminal work in social psychology and widely cited in the academic literature. TPB is a model of human behavior used to understand and predict human intention and action. It is based on the idea that human behavior results from a conscious, planned process in which people evaluate their goals and the actions available to achieve them. TPB proposes that the intention of a behavior is a relevant element in predicting a specific behavior. The intention is configured from the influence of three aspects: how attractive it is to perform a specific task, confidence in the ability to perform the task, and the presence of obstacles to doing the task. In this sense, if a person can recognize that a task is attractive to him, is confident in carrying it out, and does not consider the obstacles he would face important, it is much more likely that the intention is very large. Finally, the more intention there is, the more probability the behavior will be. TPB has been used to describe different intentions and behaviors related to education and marketing and the use of new organizational innovations. The value of the TPB lies in its potential use in generating promotion strategies for products and services.

### 3.2. Hypothesis

The relationship between variables that give rise to the tested model is presented below. Based on these relationships, the hypotheses to be evaluated are proposed.

#### 3.2.1. Effect of Ecological Concern on the Attitude

The effect of ecological concerns on attitudes towards insect, arachnid, and arthropod foods processed by 3D printing is complex and multifaceted [34–36]. On the one hand, growing awareness of the ecological impact of traditional meat production, including high levels of greenhouse gas emissions [37, 38], deforestation [39], and water use [40, 41], has led to increasing interest in alternative protein sources, including insects [42–46]. 3D printing technology to process these foods offers advantages such as reduced waste [46, 47], greater precision, and the ability to create unique shapes and textures [48, 49]. On the other hand, many people remain skeptical or disgusted at consuming insects and other arthropod-based foods, and 3D printing technology may not necessarily change these attitudes [50–52].

**Hypothesis 1 .**

Ecological concern has a positive and significant effect on attitude towards insect, arachnid, and arthropod-based foods processed by 3D printing.

#### 3.2.2. Effect of Food Safety Concern on the Attitude

When the proposal for food based on insects, arachnids, and arthropods is reviewed, the safety regarding consumption by consumers should be considered [8, 53, 54]. This concern may be due to the contamination that food could have due to toxins, bacteria or other microorganisms [8], disease transmission due to consumption of contaminated food [55], and the lack of specific standards that regulate the production of this type of food [56]. The insect-based food 3D printing process can allow these limitations and risks to be overcome, working on the quality of the product and its final physical presentation, a key aspect of ensuring the attractiveness of the food [57, 58]. 3D printing continues to advance in various industries, which is why the food industry has an excellent opportunity to adopt it to produce less traditional foods. It is very relevant that regulation facilitates the use of this technology that massifies production and can include reaching the most vulnerable populations. This could include guidelines for sourcing insects, the conditions under which they are reared, and the methods used for processing and preservation [59–61]. Consumers can also promote food safety in this industry by demanding greater transparency and accountability from manufacturers and seeking out products certified as safe and sustainable [62, 63]. While 3D printing technology may offer some potential advantages in insect-based food production, food safety remains a significant concern that must be addressed to build consumer confidence and promote growth in this industry.

**Hypothesis 2 .**

Food safety concern has a positive and significant effect on attitude towards insect, arachnid, and arthropod-based foods processed by 3D printing.

#### 3.2.3. Effect of Concern for Healthy Living on the Attitude

Concern for healthy living has been shown to influence people's attitudes towards insect, arachnid, and arthropod foods processed by 3D printing, accepting [64] and rejecting [65] the consumption. Insects and other arthropod-based foods are often considered a healthy alternative to traditional protein sources due to their high protein content, low-fat content, and high levels of micronutrients such as iron, calcium, and vitamin B12 [66, 67]. 3D printing technology in the production of insect-based foods can also positively impact the healthfulness of these products [68]. By providing greater control over the composition and texture of the final product, 3D printing can create insect-based foods that are lower in fat, salt, and other unhealthy additives [69–71]. However, it is essential to note that insect-based foods can be a healthy alternative but also present potential health risks. For example, some insects may contain allergens [72–74], and contamination of insects with pathogens or toxins may be a concern. Consumers concerned about living a healthy life can also help promote this industry's growth by seeking products certified as safe, healthy, and sustainable and demanding more regulatory agency support from manufacturers [75].

**Hypothesis 3 .**

Concern for healthy living has a positive and significant effect on attitude towards insect, arachnid, and arthropod-based foods processed by 3D printing in consumers in Panama.

#### 3.2.4. Effect of Attitude to the Purchase Intention

Some factors can influence the attitude, including cultural beliefs and traditions [76–79], personal experiences, and exposure to media and marketing [80–82]. In some cultures, insects and other arthropod-based foods have been consumed for centuries and are considered a staple food source [83–85]. In these regions, attitudes towards insect-based foods are generally positive, and 3D printing technology can be seen as a way to improve the quality, consistency, and appeal of these products. However, insects and other arthropod-based foods are not part of the traditional diet in many Western cultures, and there is often a strong aversion or disgust towards these foods [84]. In these regions, 3D printing technology may be seen as a way to make these foods more palatable, but it is unlikely to change underlying cultural attitudes. Educational campaigns [86] can help raise consumer awareness and interest in these products, while negative media coverage or food scares can have a negative impact [87]. By addressing these factors and creating a positive attitude towards these products, the insect-based food industry can significantly contribute to SDGs [88] and ensure the growth of companies dedicated to producing this type of food [89].

**Hypothesis 4 .**

Attitude towards insect, arachnid, and arthropod-based foods has a positive and significant effect on the purchase intention of insect, arachnid, and arthropod-based foods processed by 3D printing in consumers in Panama.

### 3.3. Research Model

The items for each variable are described. The model is aimed at testing the effect of ecological concern, food safety concern, and concern for healthy living on the attitude towards insect, arachnid, and arthropod-based foods. Then, the effect of attitude towards insect, arachnid, and arthropod-based foods on intention to purchase is measured. Also, it evaluates the mediating effect in the model of attitude towards insect, arachnid, and arthropod-based foods. The hypotheses, including mediation, are as follows:

**Hypothesis 5 .**

Attitude is a mediator between food safety concern and purchase intention.

**Hypothesis 6 .**

Attitude is a mediator between concern for healthy living and purchase intention.

**Hypothesis 7 .**

Attitude is a mediator between ecological concern and purchase intention.

## 4. Materials and Methods

### 4.1. Design

This study is aimed at exploring the relationships between ecological concern, food safety concern, and concern for healthy living with attitudes towards insect-based foods and how these attitudes influence purchase intention. It is a correlational and cross-sectional study. Data were obtained from Panama consumers using nonprobabilistic sampling.

### 4.2. Instrument

A questionnaire with a 5-point Likert scale was built for the current research. Four items of ecological concern were adapted from Nuttavuthisit and Thøgersen [90], Lee and Yun [91], and Rainey et al. [92]; food safety concern included four items adapted from Voon, Ngui, and Agrawal [93] and Hwang [94]. The four items for concern for healthy living were adopted from Roddy, Cowan, and Hutchinson [95]; Lee and Yun [91]; and Squires, Juric, and Bettina Cornwell [96]. Dean, Raats, and Shepherd [97] and Arvola et al. [98] adopted the two items for attitude towards insect-, arachnid-, and arthropod-based food. The instrument was developed and applied in Spanish. The original items were adapted by adjusting language and terminology to make them culturally relevant and understandable to the target population. Phrases were modified to reflect local experiences and contexts better.

### 4.3. Sample

The data was collected survey from consumers over 18 years of age between June and August 2021, using an online survey shared by WhatsApp and social media (snowball technique). The snowball technique involved contacting initial participants and asking them to recommend others who fit the study criteria. This process was repeated, gradually expanding the network of participants. By leveraging the participants' knowledge and connections, we could identify other relevant individuals, making it easier to collect data from hard-to-reach or inaccessible populations. It collected 454 answers. The data was cleaned, and 429 valid surveys were obtained. The study followed the ethical guidelines of the Declaration of Helsinki. It maintained the confidentiality of the patient's information. Nonidentifying keys were used to handle the data.

### 4.4. Data Analysis

The PLS-SEM analysis technique was used for statistical analysis for multivariate research. SMART PLS 3.3.3 software was used. The specific evaluation of the data included calculations of reliability, validity, and bootstrapping by 5000 resampling [99]. Also, the mediating effects between variables of the research model were evaluated.

## 5. Results

### 5.1. Demographic Data

The participants were 190 (44.2%) females and 239 males (55.8%). The year mean was 44.46 (SD = 14.7).

### 5.2. Reliability of Questionnaire

It was evaluated that the scales of the questionnaire focus on internal consistency.  Table 1 presents the outcomes of Cronbach's alpha, which expresses the reliability; the values were more than 0.7. Composite reliability (CR) was confirmed, with values exceeding the minimum threshold of 0.7. It was found that the total of the items exceeded 0.7, which is the minimum needed in exploratory studies.

### 5.3. Convergent and Discriminant Validity

Average variance extracted (AVE) is shown by convergent validity. Also, it evaluated the discriminant validity by the Fornell–Larcker criterion [100]. The Fornell–Larcker criterion in structural equation modeling ensures that each construct's AVE is higher than the shared variance with any other construct in the model.  Table 2 shows the fulfillment of this criterion for the variables.

### 5.4. Goodness of Fit

Table 3 shows *R* square and *R* square adjusted to evaluate the model's goodness of fit. The degree of coupling among the original data and the theoretical values calculated by regression is evaluated by goodness of fit.

### 5.5. Evaluation of the Structural Model

For the evaluation of the structural model, it used the bootstrapping technique [101], which is a statistical technique that uses resampling with replacement to estimate the distribution of a statistic in a population, especially when the sample size is small or the assumption of normality is not met. For the calculation, 5000 resampling was applied, considering *p* value < 0.05.  Table 4 shows the evaluation of the hypothesis proposed. All the hypotheses were accepted because all the effects were positive and were significant according to the *p* value (*p* < 0.001).

Table 5 shows the specific indirect effects. Hypotheses 5, 6, and 7 were accepted based on calculation because the *p* value was less than 0.05 (*p* < 0.001). In other words, ATT is a mediator between FSC and PI. Also, ATT shows that it is a mediator between CHL and PI. Finally, ATT demonstrated to be a mediator between EC and PI.

**Figure 1:**
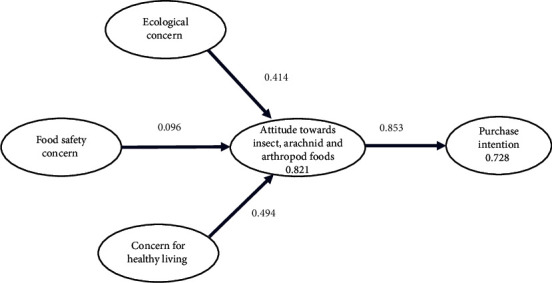
Research model evaluated.

The results verified that FSC, CH, EC, and ATT positively and significantly affect PI (Figure 1).

### 5.6. Test of Hypotheses

Table 6 shows the evaluation of the hypotheses proposed in the current study. All the hypotheses were supported by calculation.

## 6. Discussion

Like the present study (0.853; *p* < 0.001), previous studies showed the effect of attitude on purchase intention, which was described by Chang, Ma, and Chen in Taiwan [102] (0.772; *p* < 0.001); Choe, Kim, and Hwang in South Korea [103] (0.458; *p* < 0.001); and Thu Thu Aung et al. [104] in Myanmar (0.853; *p* < 0.001). Our study found a positive and significant effect of ecological concern on attitude (0.414; *p* < 0.001), unlike what was reported by Thu Thu Aung et al. [104] (0.11; *p* > 0.05). Similar to the current study (0.096; *p* < 0.001), the effect of food safety concern on attitude also was reported by Tan et al. [105] in Malaysia (0.106; *p* < 0.05). Concern about the potential negative consequences of consuming insects is expected as it is a significant disruption since insects are usually seen as carriers of toxins and sources of disease. As consumption increases and the safety of insect, arachnid, and arthropod-based foods can be confirmed, consumers could become more interested and trusting, which can improve attitudes towards these products.

Concern for healthy living showed a positive effect on food attitudes based on insects, arachnids, and arthropods. Similar results have been reported by Tan et al. [105] in Malaysia, Junges et al. [106] in Brazil, and Poortvliet [107] in the Netherlands. People may feel more inclined to eat more natural foods that can help them maintain good health. It should be noted that although these are natural inputs, industrial processing will involve preservatives. Following TPB, attitude predicts purchase intention for insect, arachnid, and arthropod-based foods. This result was found in the present research and was also reported by La Barbera [108] in Chile, Menozzi et al. [109] in Italy, and Dupont and Fiebelkorn [110] in Germany. Several strategies can be used to increase purchase intent for insect-based products, such as informing consumers about the benefits of insects as food, their high protein content, and their low environmental impact. It is also necessary to ensure insect-based products are attractively presented and offered in tasty varieties to increase their appeal to consumers. There are several ways to improve the flavor of insect foods [9], such as marinating insects in a mixture of herbs, spices, and oils, which can help give them a more intense and richer flavor. Another way is proper cooking to improve the flavor of insects; adding seasonings is also an option; that is, adding herbs, spices, and other seasonings insects can help improve their flavor. For example, lemon leaves, garlic, and paprika can be added. A specific way to enhance the appeal is to look for insect-specific recipes that can help improve their flavor and discover new flavor combinations. Texture is an important factor in food flavor. Some insects can be crushed or ground for a smooth texture, while others can be fried or roasted to create a crunchy texture [66, 111, 112].

Food safety concern may not significantly influence attitudes because participants may not be sufficiently informed about the risks. Likewise, confidence in government regulations and company security standards can be high and, therefore, cannot generate security fears. On the other hand, cost concern may take priority over safety. Personal and cultural experiences may minimize risk perception, and media exposure may not sufficiently emphasize these issues.

Companies need to promote the goodness of foods based on insects, arachnids, and arthropods; thus, using influencers and creating content on social networks can be decisive for successful promotion. In this way, partnerships can be achieved with retailers and restaurants to use fairs to help spread the flavors of these products that are often very strange to much of the population. Several significant companies sell insect-based foods. Some of the best-known include Aspire Food Group and Hexa Foods (https://www.aspirefg.com), which specialize in producing and marketing maguey worm foods. Entomo Farms (https://entomofarms.com) focuses on producing insect-based foods, such as crickets, escamoles, and cotton larvae.

In this research, TPB [33] shows that attitude towards insects, arachnids, and arthropods strongly predicts intention to purchase these kinds of products. The production and consumption of food based on insects, arachnids, and arthropods can be an important solution to achieve SDGs through reduced environmental impact: Insect-based food production requires fewer resources and emits fewer greenhouse gases than animal-based meat production. It is also relevant to mention that better food efficiency can be achieved since insects are a very efficient source of protein, requiring less feed and water to produce a similar amount of protein than animals. An important aspect is food security, which was negatively impacted due to COVID-19 [113–115]. Precisely, the production and consumption of these foods can contribute to food security because insects can be cultivated in various environments and climates, making them a promising option for food security in poorer regions of the world. This chance that suppliers of this raw material can achieve can reduce poverty by creating employment opportunities and income generation in local communities.

The outcomes showed that the questionnaire applied was valid and reliable. Future studies may use the questionnaire and the model to test the effect of its variables and be able to incorporate some variables that can help complete the understanding of the purchase intention of foods based on insects, arachnids, and arthropods. It will be relevant for future studies to be carried out in different regions to compare preferences among consumers and find differences and similarities.

## 7. Conclusions

The study has shown that ecological concern and concern for healthy living have a positive and significant effect on the attitude towards insect, arachnid, and arthropod-based foods. It was also possible to show that the food safety concern did not have a positive and significant effect on the attitude towards insect, arachnid, and arthropod-based foods. Likewise, the attitude towards insect, arachnid, and arthropod-based foods showed a positive and significant effect on purchase intention. On the one hand, it was evident that the attitude towards insect, arachnid, and arthropod-based foods is a mediator between ecological concern and purchase intention. The mediation of attitude towards insect, arachnid, and arthropod-based foods between food safety concern of healthy living and purchase intention was also evident. Finally, it was not demonstrated that the attitude towards insect, arachnid, and arthropod-based foods is a mediating variable between food safety concern and purchase intention.

## Figures and Tables

**Table 1:** Construct validity using PLS-SEM.

**Scale item**	**Factorial weight**	**CR**	**AVE**
*Ecological concern (I consider that….)*		0.863	0.711
… food based on insects, arachnids, or arthropods can be produced for human consumption	0.905		
… food based on insects, arachnids, or arthropods can be environmentally friendly	0.802		
… food based on insects, arachnids, or arthropods should be produced in such a way that they do not feel pain	0.759		
… food based on insects, arachnids, or arthropods could be safer for the environment than conventional food	0.897		
*Food safety concern (I am concerned…)*		0.907	0.790
… that most foods based on insects, arachnids, or arthropods contain chemical residues	0.889		
… about the amount of artificial additives and preservatives in foods based on insects, arachnids, or arthropods	0.946		
… about the quality and safety of food based on insects, arachnids, or arthropods	0.970		
… about how foods based on insects, arachnids, or arthropods could be processed	0.730		
*Concern for healthy living (I believe that foods…)*		0.906	0.780
… based on insects, arachnids, or arthropods could have higher levels of vitamins and nutrients than foods available	0.908		
… based on insects, arachnids, or arthropods could be consumed if they are processed and printed in a 3D food printer	0.814		
… based on insects, arachnids, or arthropods could contain higher amounts of protein than traditional foods	0.923		
… based on insects, arachnids, or arthropods to ensure good health would be chosen by me if they were available	0.882		
*Attitude towards insect, arachnid, and arthropod-based foods (I believe that buying food based on …)*		0.934	0.938
… insects, arachnids, or arthropods instead of conventional food could guarantee food security	0.966		
… insects, arachnids, or arthropods processed by 3D printing instead of conventional food would make me feel satisfied	0.971		
*Purchase intention*		0.947	0.866
I intend to buy insect, arachnid, or arthropod-based 3D-printed food if it were available	0.950		
If insect, arachnid, or arthropod-based foods were available in stores, I would buy them	0.933		
I intend to buy foods based on insects, arachnids, or arthropods even if they cost more than traditional foods	0.852		
I intend to purchase food based on insects, arachnids, or arthropods processed by 3D printing	0.982		

**Table 2:** Discriminant validity.

**Variable**	**ATT**	**CHL**	**EC**	**FSC**	**PI**
ATT	0.969				
CHL	0.874	0.883			
EC	0.845	0.821	0.843		
FSC	0.413	0.420	0.266	0.889	
PI	0.853	0.824	0.889	0.264	0.931

Abbreviations: ATT = attitude towards insect, arachnid, and arthropod-based foods; CHL = concern of healthy living; EC = ecological concern; FSC = food safety concern; PI = purchase intention.

**Table 3:** *R* square and *R* square adjusted.

**Scale**	**R** ** square**	**R** ** square adjusted**
ATT	0.821	0.819
PI	0.728	0.728

Abbreviations: ATT = attitude towards insect, arachnid, and arthropod-based foods; PI = purchase intention.

**Table 4:** Bootstrapping results.

**H**	**Hypothesis**	**Beta**	**Mean**	**SD**	**T** ** value**	**p** ** value**
H1	EC ➔ ATT	0.414	0.414	0.032	12.843	*p* < 0.001
H2	FSC ➔ ATT	0.096	0.098	0.023	4.225	*p* < 0.001
H3	CHL ➔ ATT	0.494	0.493	0.030	16.540	*p* < 0.001
H4	ATT ➔ PI	0.853	0.853	0.011	74.581	*p* < 0.001

Abbreviations: ATT = attitude towards insect, arachnid, and arthropod-based foods; CHL = concern of healthy living; EC = ecological concern; FSC = food safety concern; PI = purchase intention; SD = standard deviation.

**Table 5:** Specific indirect effects.

**Scale**	**Original sample**	**Sample mean**	**SD**	**T** ** value**	**p** ** value**
H5: FSC ➔ ATT ➔ PI	0.082	0.083	0.019	4.282	*p* < 0.001
H6: CHL➔ ATT ➔ PI	0.422	0.421	0.025	16.619	*p* < 0.001
H7: EC ➔ ATT ➔ PI	0.353	0.353	0.029	12.104	*p* < 0.001

Abbreviations: ATT = attitude towards insect, arachnid, and arthropod-based foods; CHL = concern of healthy living; EC = ecological concern; FSC = food safety concern; PI = purchase intention; SD = standard deviation.

**Table 6:** Test of hypotheses.

**Hypothesis**	**Outcome**	**Support**
H1: EC has a positive and significant effect on ATT	0.414 (*p* < 0.001)	Yes
H2: FSC has a positive and significant effect on ATT	0.096 (*p* < 0.001)	Yes
H3: CHL has a positive and significant effect on ATT	0.494 (*p* < 0.001)	Yes
H4: ATT has a positive and significant effect on PI	0.853 (*p* < 0.001)	Yes
H5: ATT is a mediator between FSC and PI	0.082 (*p* < 0.001)	Yes
H6: ATT is a mediator between CHL and PI	0.422 (*p* < 0.001)	Yes
H7: ATT is a mediator between CE and PI	0.353 (*p* < 0.001)	Yes

Abbreviations: ATT = attitude towards insect, arachnid, and arthropod-based foods; CHL = concern of healthy living; EC = ecological concern; FSC = food safety concern; PI = purchase intention; SD = standard deviation.

## Data Availability

The data that support the findings of this study are available on request from the authors.
